# Coding-Complete Genome Sequence of Swine Influenza Virus Isolate A/Swine/Karaganda/04/2020 (H1N1) from Kazakhstan

**DOI:** 10.1128/MRA.00786-21

**Published:** 2021-09-30

**Authors:** Nailya G. Klivleyeva, Nuray S. Ongarbayeva, Ilya S. Korotetskiy, Tatiana I. Glebova, Nurbol T. Saktaganov, Mira G. Shamenova, Baiken B. Baimakhanova, Alexandr B. Shevtsov, Asylulan Amirgazin, Vladimir E. Berezin, Richard J. Webby

**Affiliations:** a The Research and Production Center for Microbiology and Virology, Almaty, Kazakhstan; b Faculty of Biology and Biotechnology, al-Farabi Kazakh National University, Almaty, Kazakhstan; c Scientific Center for Anti-Infectious Drugs, Almaty, Kazakhstan; d National Center for Biotechnology, Nur Sultan, Kazakhstan; e St. Jude Children's Research Hospital, Memphis, Tennessee, USA; Queens College CUNY

## Abstract

Here, we report the coding-complete genome sequence of a clinical sample of influenza virus obtained from a pig at a livestock farm in Karaganda, Central Kazakhstan, during a pig study in 2020. Isolate A/Swine/Karaganda/04/2020 (H1N1) belongs to clade 1A.3.2.2 lineage 1A, which includes the 2009 H1N1 pandemic strains.

## ANNOUNCEMENT

Influenza A viruses (IAV) belong to the genus *Alphainfluenzavirus* in the family *Orthomyxoviridae* and cause one of the most important respiratory diseases in pigs as well as humans (1–3). An important role in the emergence of pandemic strains can be played by genetic reassortment between human and avian influenza viruses in pigs, since swine are equally susceptible to both human and avian influenza viruses (4).

In 2020 at a livestock farm in Karaganda, Kazakhstan, nasopharyngeal swabs were collected from pigs 2 to 5 months of age. All manipulations with animals were approved by the Institutional Animal Care and Use Committee from the Research and Production Center for Microbiology and Virology, Almaty, Kazakhstan. Viral RNA was extracted from a single sample using the PureLink viral RNA/DNA minikit (Invitrogen). The IAV segments were amplified by reverse transcriptase PCR (RT-PCR) using the protocol proposed by Zhou et al. (5). Library preparation was performed using the Nextera DNA Flex library prep kit, and sequencing was performed using the MiSeq reagent kit v2 (Illumina) according to the manufacturer’s protocols. For A/Swine/Karaganda/04/2020 (H1N1), a total of 623,688 reads were generated with an average length of 255 nucleotides. Default parameters were used for all the following analytical software tools. After read quality trimming using UGENE v39.0 (6), the reads were assembled *de novo* using SPAdes v3.15.2. The resulting contigs were used to conduct a BLASTn search for reference sequences in the GenBank database. The search was restricted to only Influenza A virus sequences (taxid, 11320). Mapping against the 8 segments of the reference strains was performed using BWA software (Table 1).

**TABLE 1:** Reference sequences for all gene segments of isolate A/Swine/Karaganda/04/2020 (H1N1)

Segment	Reference strain	Identity at nucleotide level (%)	GenBank accession no.
1	A/Baltimore/R0252/2018 (H1N1)	99.19	MH637811.1
2	A/Missouri/51/2017 (H1N1)	99.35	MH083791.1
3	A/Arizona/75/2017 (H1N1)	99.41	MH083356.1
4	A/USA/SC7097/2018 (H1N1)	99.10	MK168543.1
5	A/Baltimore/R0264/2018 (H1N1)	99.42	MH637700.1
6	A/Baltimore/R0252/2018 (H1N1)	98.29	MH637715.1
7	A/Swine/France/43-180020/2018 (H1N1)	99.22	MT379017.1
8	A/Baltimore/R0258/2018 (H1N1)	99.21	MH637476.1

The genome assembly of A/Swine/Karaganda/04/2020 (H1N1) was 13,611 bp long (average coverage, 40×). An influenza virus sequence annotation tool (7) was used to automatically generate annotations for the 8 segments of the strain.

**FIG 1:**
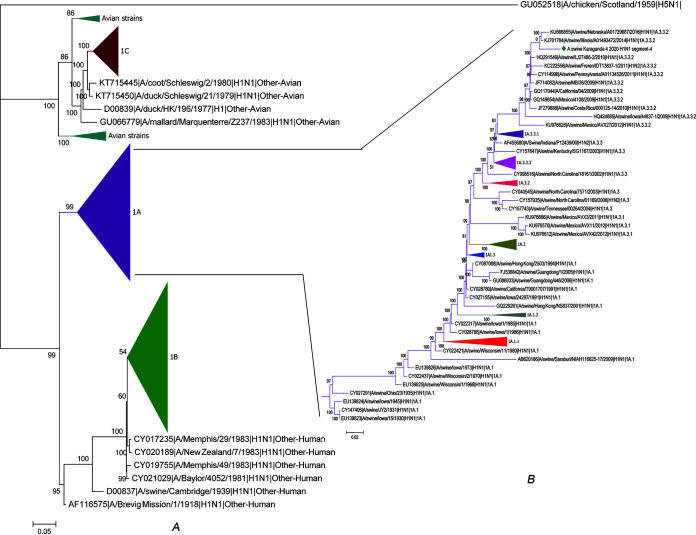
(A) Total phylogenetic tree with global swine H1 clade classification. (B) Subtree of clade 1A influenza hemagglutinin (HA) genomes. Strain A/Swine/Karaganda/04/2020 (H1N1) is marked with a rhombus.

Using the Swine H1 Clade Classification Tool from the Influenza Research Database (IRD) (8), the Kazakh isolate was determined to belong to clade 1A.3.2.2 lineage 1A. This clade has a wide global distribution, which includes the 2009 H1N1 pandemic strains. The phylogenetic tree for the hemagglutinin gene was constructed using the likelihood criterion for optimization, model HKY85, and with 500 replicas for bootstrapping using the IRD Web server. The tree was downloaded in a Newick format file, and MEGA7 (9) was used for visualization, collapsing, and coloring of some of the branches (Fig. 1).

The obtained genome sequences will be used to study virus circulation and genetic variation for inducing the reversal of drug sensitivity (10). Swine strains can be reintroduced into the human population after a certain period and cause a pandemic, as illustrated by the influenza A(H1N1)pdm09 virus.

### Data availability.

The data for the 8 segments of strain A/Swine/Karaganda/04/2020 (H1N1) are available at NCBI under GenBank accession numbers MZ363969.1 to MZ363976.1. The raw sequence reads can be accessed through the Sequence Read Archive (SRA) database under accession number SRR15011445 and BioProject accession number PRJNA742842.
